# Preoperative hemoglobin A1c and minimally invasive lumbar spine surgery: is it as critical as we think

**DOI:** 10.1007/s00701-025-06686-2

**Published:** 2025-11-08

**Authors:** Yifei Sun, Sasha Howell, Lucia D. Juarez, B. Grey Vandeberg, Nicholas M. B. Laskay, Jovanna Tracz, James Mooney, Jakub Godzik

**Affiliations:** 1 Heersink School of Medicine, University of Alabama at Birmingham, Birmingham, AL USA; 2 Department of Neurosurgery, University of Alabama at Birmingham, Birmingham, AL USA; 3 Division of General Internal Medicine and Population Science, Heersink School of Medicine, University of Alabama at Birmingham, Birmingham, AL USA; 4 Department of Neurosurgery, Virginia Commonwealth University, Richmond, VA USA

**Keywords:** HbA1c, Readmissions, Reoperation, Minimally invasive, Complications

## Abstract

**Background:**

Minimally invasive approaches to lumbar spine surgery are increasingly popular. Current guidelines highlight the importance of preoperative HbA1c in optimizing spine surgery outcomes. However, the role of preoperative HbA1c in minimally invasive lumbar spine surgery remains unclear.

**Objectives:**

We sought to assess the association of HbA1c with readmissions, reoperations, and complications following minimally invasive lumbar spine surgery.

**Methods:**

We retrospectively reviewed all adult patients at a single institution from 2011 to 2023 who underwent minimally invasive lumbar decompression or decompression with instrumented fusion using CPT and ICD9/10 codes. Multivariate logistic regressions were performed to assess the effect of high HbA1c on readmissions and reoperations.

**Results:**

In total, 1013 [median age 64 (IQR 54–71)] patients met the inclusion criteria. The median preoperative HbA1c was 5.99% (IQR 5.62 – 6.39). Upon multivariate regression analysis adjusting for frailty, socioeconomic status, and other confounders, patients with high HbA1c (> 7.1) had increased odds of unplanned readmission within 90 days (OR 2.02, 95% CI 1.10– 3.56, p = 0.019) and reoperation within 90 days (OR 2.82, 95%CI 1.14–6.31) of the index operation. Patients with high HbA1c also had increased odds of requiring reoperation due to persistent symptoms (OR 2.9, 95%CI 0.91–7.87, p = 0.048). After propensity score matching, patients with high HbA1c also had prolonged hospital lengths of stay (1.32 days vs 1.24 days, p = 0.006), post operative UTI (4.7% vs 0.9%, p = 0.034).

**Conclusions:**

Our results suggest high preoperative HbA1C may be associated with increased rates of readmission and reoperation following minimally invasive lumbar spine surgery. Preoperative HbA1C control may be indicated for surgical optimization in minimally invasive lumbar spine surgery.

**Supplementary Information:**

The online version contains supplementary material available at 10.1007/s00701-025-06686-2.

## Introduction

Spine surgery is one of the most common procedures performed nationwide, with utilization rates trending upwards in recent years given our increasingly aging populatio [16]. In the era of value based care, it is increasingly important to optimize surgical outcomes and reduce complication rates and hospital readmissions. Much of literature has focused on the value of comorbidity control in preventing readmissions, with several studies demonstrating the value of optimizing comorbidities in ensuring good surgical outcomes [1, 3, 4, 36]. One of the main focuses of preoperative optimization lay in the optimization of pre-operative hemoglobin A1c (HbA1c) [11].

Often a sign of poorly controlled diabetes, elevated pre-operative HbA1c has been shown to predict poor outcomes following spine surgery, with studies highlighting an association with increased rates of complication, infections, readmission rates, and reoperation rates [28, 29, 31]. Other studies have demonstrated the association of pre- and perioperative HbA1c with increased rates of both deep and superficial surgical site infections (SSI) [12]. Other studies have shown that poor HbA1c is associated with failure to reach minimally clinically important differences (MCID) in a variety of validated patient reported outcome (PROs) and pain scales as well [9].

However, despite the abundance of literature describing the role of HbA1c in open spine surgery, the role of HbA1c optimization in minimally invasive (MIS) lumbar spine surgery remains poorly understood. MIS approaches to lumbar spine surgery are increasingly popular, offering benefits including faster recoveries, shorter hospital lengths of stay, and lower blood loss when compared to open lumbar spine approaches.[18, 20] MIS approaches often require less tissue dissection and offer smaller wounds. Given the less invasive nature of these procedures, the risk imparted by high HbA1c may not be as pronounced when compared to open procedures. Thus, it remains unknown whether strict HbA1c control is necessary in MIS cases and given the potential mechanism of action, worth critically evaluating.

Given the paucity of evidence emphasizing the importance of preoperative HbA1c optimization regarding the effect of HbA1c on MIS spine outcomes, we sought to investigate this question. Our primary objective was to elucidate the association of high preoperative HbA1c with unplanned readmissions and infection/wound breakdown after minimally invasive lumbar spine surgery. Our secondary outcomes include to uncover the association between preoperative HbA1c and complications, and hospital lengths of stay. In doing so, we hope to better understand the effect of preoperative HbA1c on MIS spine surgery outcomes.

## Methods

This single center study was designed as a retrospective cohort review with Institutional Review board approval (IRB-300009309). This article was written in compliance with the STROBE (Strengthening the Reporting of Observational Studies in Epidemiology) guidelines [33].

We identified all patients who underwent MIS lumbar spine surgery from 2011 to 2023 using CPT and ICD9/10 codes. Inclusion criteria were adult patients (≥ 18 years) undergoing elective MIS lumbar spine surgery. All patients underwent surgical decompression or decompression with instrumented fusion via a minimal exposure tubular retractor system. Patients undergoing minimally invasive anterior approaches or percutaneous screw placements were also included. Patients who underwent surgery for traumatic indications were excluded. Informed consent was not sought due to the retrospective nature of this study.

### Variables

Study variables were collected via review of the electronic medical record. Variables included were age at time of surgery categorized according to standard groups (< 65, 65–75, and ≥ 75), race which was grouped as white, African American, and other), gender (Male or Female), preoperative smoking status, and insurance status which was categorized as private (Blue Cross/Blue shield, Aetna, etc.), public (Medicare, Medicaid), or indigent/self-pay [5, 21]. Preoperative HbA1c was collected, and high HbA1c was categorized as > 7.1% based on analysis identifying optimal cutoff for maximum predictive power for 90 day readmission (Supplemental Digital Content, Fig. 1S). Frailty was assessed via the hospital frailty risk score (HFRS) and calculated according to validated methods [8]. Overall socioeconomic status was assessed using Area Deprivation Index (ADI), a well validated measure of neighborhood socioeconomic status. ADI was retrieved from the Neighborhood Atlas dataset produced by the Center for Health Disparities Research at the University of Wisconsin School of Medicine and Public Health, with higher ADI indicating a higher level of socioeconomic disparity [14, 27]. High ADI was defined as being the top quartile of national ADI percentile. Further details regarding variable outcomes definition can be found in the supplement (Supplementary Digital Content, Supplemental Methods).

### Statistical analysis

Categorical, binary, and ordinal variables were reported as counts and percentages, while continuous variables were reported as the median and interquartile range (IQR). Univariable comparison analysis was performed via utilizing the one-way analysis of variance (ANOVA), Pearson’s chi-squared test, Wilcoxon rank sum test, or Fisher’s exact test.

Multivariate logistic regression was additionally conducted on primary outcomes to determine the independent effect of high preoperative HbA1c and to serve as additional sensitivity analysis. Odds ratios (OR) with 95% confidence intervals (CIs) are reported. Multicollinearity of the multivariate logistic regression models was assessed using the variance inflation factor (VIF) (Supplemental Digital Content,Table 1S-2S) [32]. To serve as sensitivity analysis, we conducted propensity score matching, matching for age, neighborhood deprivation, fusion vs decompression alone, gender, number of levels operated on, race, preoperative smoking status, insurance status, ASA class, frailty status, and BMI. Matching was done via the R package *Matchit* with nearest neighbor 4:1 matching with a 0.5 caliper to maximize sample size and minimize any bias potentially introduced from pruning [22]. Match quality was assessed by visual inspection of a plot of the absolute distances between covariates after successful matching and via ensuring the adjusted mean differences between groups was < 0.1 for all variables (Supplemental Digital Content**, **Fig. 2S). There was a high degree of missingness for preoperative HbA1c values (43%). Given the potential to introduce significant selection bias if complete case analysis was utilized, multiple imputations using the full dataset using the missForest technique was performed, with low normalized root mean squared error (NRMSE) (2E-6) and a miss-classification rate of 3%, reflecting highly accurate imputation (Supplemental Digital Content, Table 3S**, **Fig. 3S**) **[26]. Statistical significance was set at α = 0.05**,** and all tests for significance were two-sided. All statistical analyses were performed using R (version 4.3.1, R Foundation for Statistical Computing, Vienna, Austria) [30].

## Results

### Demographics

In total, 1013 patients who underwent MIS lumbar spine decompression with or without fusion were included for analysis. Most of the procedures were posterior (88%) with 8.8% being anterior, and 4.0% combined. The median age at time of surgery was 64 (IQR 54 −71). Fifty-four percent of the cohort was male and 20% were black, with 31% and 18% of the cohort being former or current smokers at time of index operation, respectively. The median HbA1c 5.99% (5.62–6.4) The majority of procedures involved a single interbody space (67%) with 32% including 2–3. Of the cohort, 35% had a history of diabetes mellitus, with 13% having diabetes with complications. The results of this analysis can be found in Table 1.

**Table 1:** Patient characteristics and Demographics

Characteristic	Overall
	N = 1,013^*1*^
Principle Indication	
Degenerative Lumbar Spondylosis	645 (64%)
Degenerative Lumbar Spondylolisthesis	44 (4.3%)
Disc Herniation	130 (13%)
Lumbar Stenosis	178 (18%)
Lumbar Radiculopathy	15 (1.5%)
Other	1 (< 0.1%)
Pre op HbA1C	5.99 (5.62, 6.39)
Age	64 (54, 71)
< 65	532 (53%)
65–75	335 (33%)
> 75	146 (14%)
Gender	
Female	465 (46%)
Male	548 (54%)
Race	
White	768 (76%)
Black	199 (20%)
Other	46 (4.5%)
Smoking Status	
Never	518 (51%)
Former	309 (31%)
Current	186 (18%)
HFRS Frailty score	
Low Risk	638 (63%)
Intermediate Risk	203 (20%)
High Risk	172 (17%)
Area Deprivation Index (National Percentile)	63 (43, 81)
ASA class	
1	4 (0.4%)
2	181 (18%)
3	794 (78%)
4	34 (3.4%)
Preoperative Diabetes	350 (35%)
Preoperative Diabetes with Complications	130 (13%)
BMI (kg/m^2^)	30 (27, 35)
Normal weight	144 (14%)
Underweight	10 (1.0%)
Overweight	330 (33%)
Obese	529 (52%)
Total Intervertebral Levels	
1	676 (67%)
2	324 (32%)
3 +	13 (1.3%)
Insurance Type	
Private	316 (31%)
Public	668 (66%)
Indigent/Self Pay	29 (2.9%)
Surgical Approach	
Anterior	89 (8.8%)
Combined	41 (4.0%)
Posterior	883 (87%)

^*1*^ n (%); Median (Q1, Q3)

### Univariate comparison

**Fig. 1:**
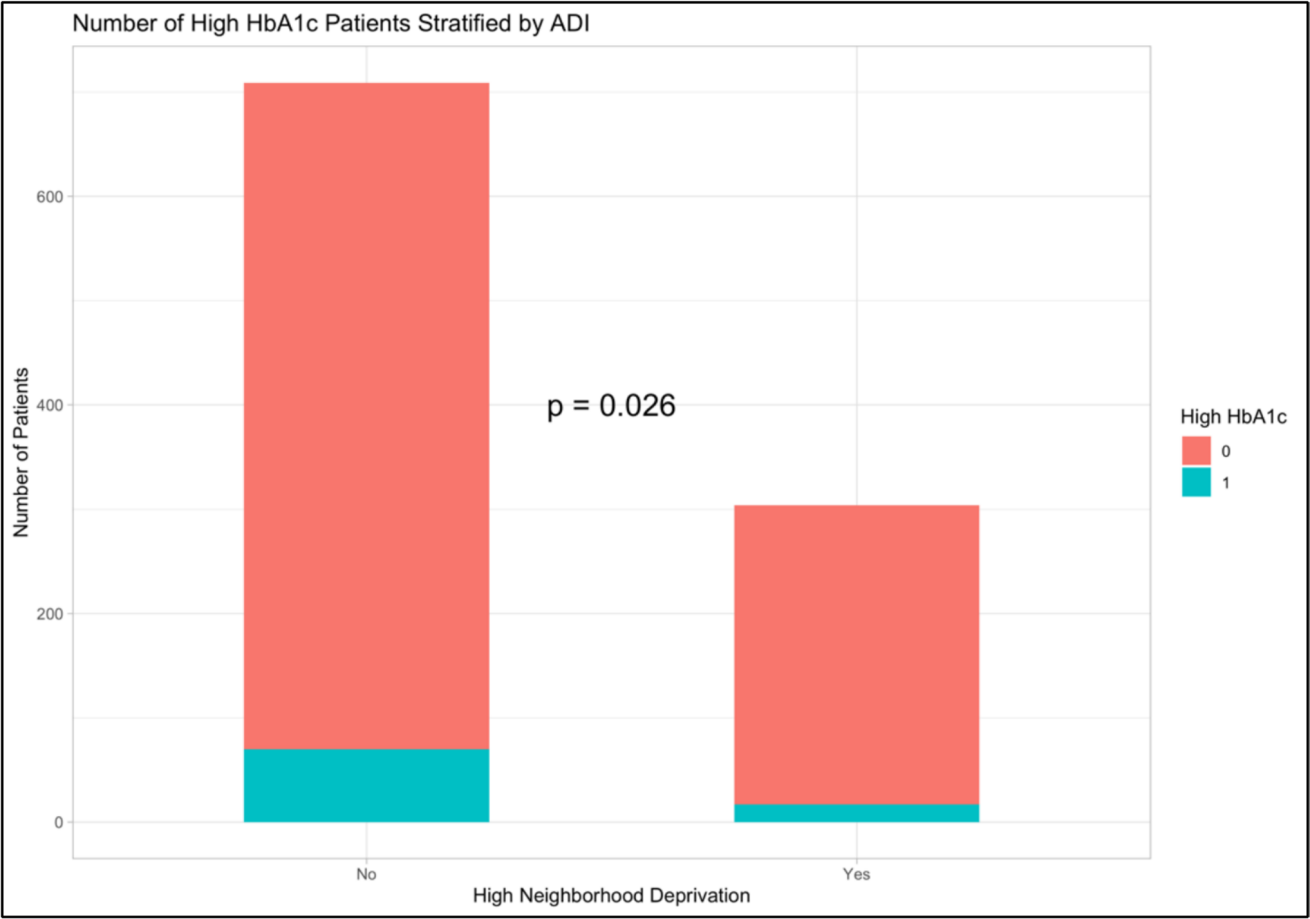
HbA1c category by Neighborhood Deprivation

Of the cohort, 87 (9.6%) of patients having an HbA1c above 7.1% before surgery. In bivariate comparison between patients with high and low HbA1C, patients with high HbA1c and were more likely to be socioeconomically disadvantaged (31% vs 20%, p = 0.026), and had higher ASA class at time of surgery (p = 0.002) (Fig. 1, Table 2).

**Table 2:** Comparison by HbA1c

Characteristic	HbA1c		
	No	Yes	p-value^*2*^
	N = 926^*1*^	N = 87^*1*^	
Principle Indication			0.3
Degenerative Lumbar Spondylosis	587 (63%)	58 (67%)	
Degenerative Lumbar Spondylolisthesis	40 (4.3%)	4 (4.6%)	
Disc Herniation	214 (23%)	13 (15%)	
Lumbar Stenosis	70 (7.6%)	11 (13%)	
Lumbar Radiculopathy	14 (1.5%)	1 (1.1%)	
Other	1 (0.1%)	0 (0%)	
Age	65 (55, 71)	60 (51, 67)	0.005
< 65	471 (51%)	61 (70%)	
65–75	319 (34%)	16 (18%)	
> 75	136 (15%)	10 (11%)	
Gender			0.3
Female	430 (46%)	35 (40%)	
Male	496 (54%)	52 (60%)	
Race			0.04
White	710 (77%)	58 (67%)	
Black	178 (19%)	21 (24%)	
Other	38 (4.1%)	8 (9.2%)	
Smoking Status			> 0.9
Never	473 (51%)	45 (52%)	
Former	284 (31%)	25 (29%)	
Current	169 (18%)	17 (20%)	
HFRS Frailty score			0.3
Low Risk	590 (64%)	48 (55%)	
Intermediate Risk	181 (20%)	22 (25%)	
High Risk	155 (17%)	17 (20%)	
ASA class			
1	4 (0.4%)	0 (0%)	0.002
2	177 (19%)	4 (4.6%)	
3	715 (77%)	79 (91%)	
4	30 (3.2%)	4 (4.6%)	
Preoperative Diabetes	272 (29%)	78 (90%)	< 0.001
Preoperative Diabetes with Complications	81 (8.7%)	49 (56%)	< 0.001
BMI (kg/m^2^)	30 (26, 35)	34 (30, 39)	< 0.001
Normal weight	141 (15%)	3 (3.4%)	
Underweight	10 (1.1%)	0 (0%)	
Overweight	310 (33%)	20 (23%)	
Obese	465 (50%)	64 (74%)	
Total Intervertebral Levels			0.7
1	615 (66%)	61 (70%)	
2	298 (32%)	26 (30%)	
3 +	13 (1.4%)	0 (0%)	
Insurance Type			> 0.9
Private	290 (31%)	26 (30%)	
Public	609 (66%)	59 (68%)	
Indigent/Self Pay	27 (2.9%)	2 (2.3%)	
Surgical Approach			0.7
Anterior	80 (8.6%)	9 (10%)	
Combined	39 (4.2%)	2 (2.3%)	
Posterior	807 (87%)	76 (87%)	
Post op Complications	124 (13%)	14 (16%)	0.5
CSF-leak/Pseudomeningocele	26 (2.8%)	3 (3.5%)	0.7
Pneumonia	5 (0.5%)	0 (0%)	> 0.9
Respiratory Failure	8 (0.9%)	0 (0%)	> 0.9
Sepsis	10 (1.1%)	2 (2.3%)	0.3
Cerebrovascular	9 (1.0%)	1 (1.1%)	0.6
DVT/PTE	9 (1.0%)	0 (0%)	> 0.9
Hematoma	10 (1.1%)	0 (0%)	> 0.9
Delirium	12 (1.3%)	0 (0%)	0.6
Bowel Ileus	39 (4.2%)	4 (4.7%)	0.8
Urinary Incontinence	9 (1.0%)	1 (1.2%)	0.6
Urinary tract Infection	15 (1.6%)	4 (4.7%)	0.071
Myocardial Infarction	4 (0.4%)	2 (2.3%)	0.086
Surgical Site Infection	7 (0.8%)	1 (1.1%)	0.5
Wound Breakdown	15 (1.6%)	3 (3.5%)	0.2
Hardware failure	6 (0.7%)	0 (0%)	> 0.9
AKI	9 (1.0%)	3 (3.5%)	0.075
Other	1 (0.1%)	0 (0%)	> 0.9
Readmit 30 days	63 (6.8%)	5 (5.7%)	0.7
Medical	19 (2.1%)	2 (2.3%)	0.7
Infection/Wound	5 (0.5%)	1 (1.1%)	0.4
Surgical	39 (4.2%)	2 (2.3%)	0.6
Readmit 90 days	103 (11%)	18 (21%)	0.009
Medical	43 (4.6%)	4 (4.6%)	> 0.9
Infection/Wound	5 (0.5%)	2 (2.3%)	0.12
Surgical	63 (6.8%)	12 (14%)	0.017
Reoperated within 90 days	36 (3.9%)	8 (9.2%)	0.046
Hospital Length of Stay	1.26 (1.00, 1.50)	1.33 (1.00, 2.00)	0.013
Discharge Category			0.7
Home	775 (84%)	70 (80%)	
Home Health	107 (12%)	12 (14%)	
I/P Rehab	34 (3.7%)	4 (4.6%)	
SNF Nursing Home	10 (1.1%)	1 (1.1%)	
Total Reasons for Reoperation 90 days			
CSF/Pseudomeningocele	14 (1.5%)	3 (3.4%)	0.2
Persistent Symptom/Neurologic	19 (2.1%)	5 (5.7%)	0.048
Wound Revision	4 (0.4%)	0 (0%)	> 0.9
Other	1 (0.1%)	0 (0%)	> 0.9

^*1*^ Median (Q1, Q3); n (%)

^*2*^ Wilcoxon rank sum test; Pearson’s Chi-squared test; Fisher’s exact test

### Logistic regression analysis

**Fig. 2:**
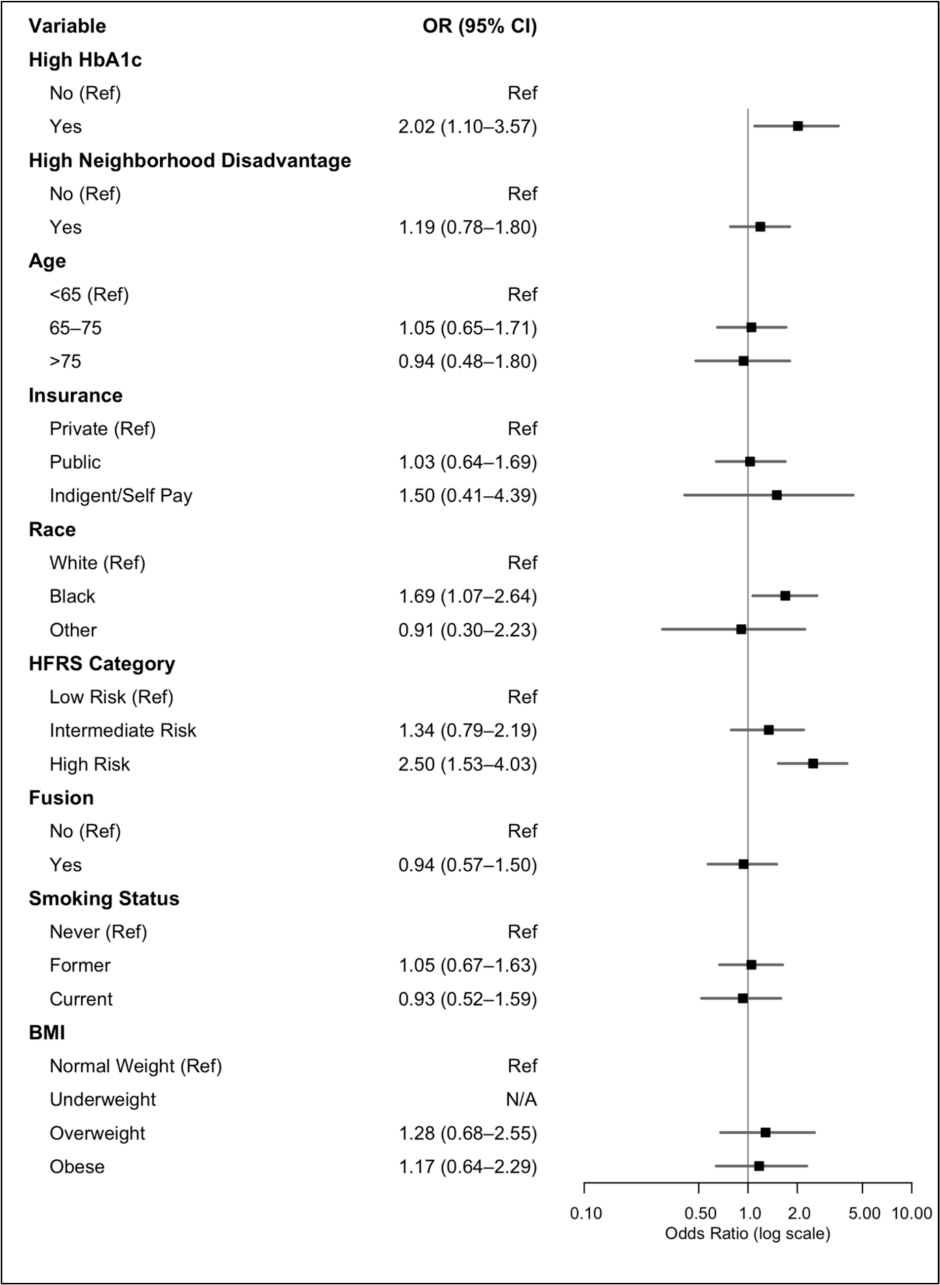
Forest plot for multivariate logistic regression for 90-day readmission

**Fig. 3:**
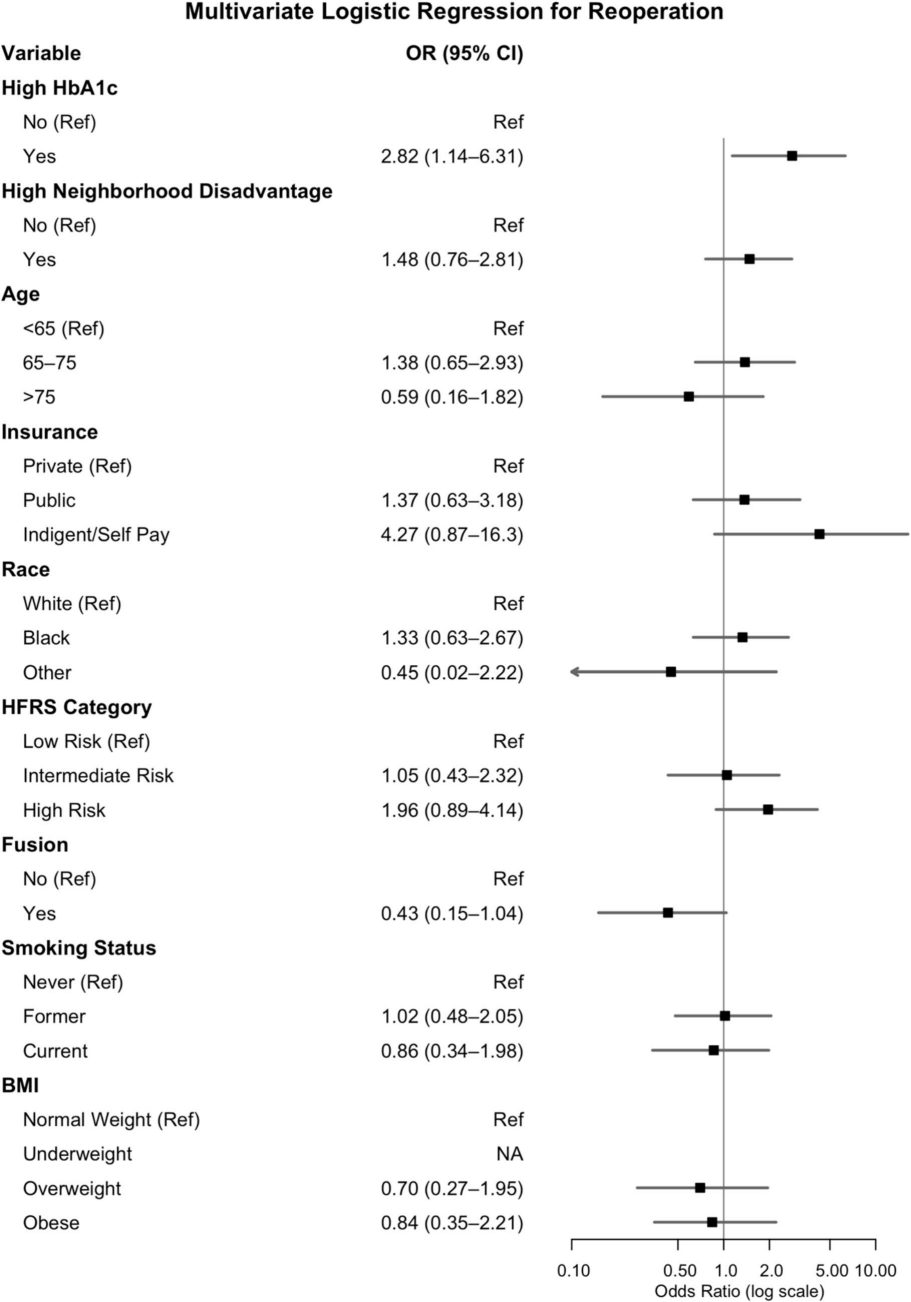
Forest plot for multivariate logistic regression for 90-day Reoperation

In multivariate logistic regression analysis, patients with high HbA1c (OR 2.02, 95%CI 1.10–3.57), patients of black race (OR 1.69, 95%CI 1.07–2.64), and patients who fell into the high-risk category of frailty (OR 2.50, 95%CI 1.53–4.03) had increased odds of 90-day readmission (Fig. 2). In multivariate logistic regression analysis, patients with high HbA1c (OR 2.82, 95%CI 1.14–6.31), and patients who were uninsured (OR 4.27, 95%CI 0.87–16.3) had increased odds of 90-day reoperation (Fig. 3). In subgroup analysis, patients with high Hba1c had independently increased odds of reoperation within 90 days due to persistent or recurrent symptoms (Supplemental Digital Content,Fig. 3S).

### Propensity score analysis

After propensity score matching, 86 patients with high preoperative HbA1c were successfully matched to 336 controls, with baseline characteristics selected for matching balanced across the two groups. The standardized distance between the two groups decreased significantly after matching (Supplemental Digital Content, Fig. 1S).

Patients with high HbA1c were more likely to be readmitted within 90 days (21% vs 11%, p = 0.011), reoperation within 90 days (9.3% vs 3.6%, p = 0.041) and had prolonged length of stay compared to matched controls (1.32 vs 1.24 days, p = 0.006). These patients also had increased risk of post operative urinary tract infection. The results of this analysis can be found in Table 3.

**Table 3:** Propensity Score Matched Analysis

Characteristic	HbA1c		
	No	Yes	p-value^*2*^
	N = 336^*1*^	N = 86^*1*^	
Post op Complications	42 (13%)	14 (16%)	0.5
CSF-leak/Pseudomeningocele	10 (3.0%)	3 (3.5%)	0.7
Pneumonia	2 (0.6%)	0 (0%)	> 0.9
Respiratory Failure	3 (0.9%)	0 (0%)	> 0.9
Sepsis	2 (0.6%)	2 (2.4%)	0.2
Cerebrovascular	2 (0.6%)	1 (1.2%)	0.5
DVT/PTE	3 (0.9%)	0 (0%)	> 0.9
Hematoma	3 (0.9%)	0 (0%)	> 0.9
Delirium	7 (2.1%)	0 (0%)	0.4
Bowel Ileus	14 (4.2%)	4 (4.7%)	0.8
Urinary Incontinence	1 (0.3%)	1 (1.2%)	0.4
Urinary tract Infection	3 (0.9%)	4 (4.7%)	0.034
Myocardial Infarction	2 (0.6%)	2 (2.4%)	0.2
Surgical Site Infection	2 (0.6%)	1 (1.2%)	0.5
Wound Breakdown	6 (1.8%)	3 (3.5%)	0.4
Hardware failure	2 (0.6%)	0 (0%)	> 0.9
AKI	3 (0.9%)	3 (3.5%)	0.1
Readmit 30 days	21 (6.3%)	5 (5.8%)	0.9
Medical	9 (2.7%)	2 (2.3%)	> 0.9
Infection/Wound	2 (0.6%)	1 (1.2%)	0.5
Surgical	10 (3.0%)	2 (2.3%)	> 0.9
Readmit 90 days	36 (11%)	18 (21%)	0.011
Medical	16 (4.8%)	4 (4.7%)	> 0.9
Infection/Wound	2 (0.6%)	2 (2.3%)	0.2
Surgical	22 (6.5%)	12 (14%)	0.024
Reoperated within 90 days	12 (3.6%)	8 (9.3%)	0.041
Hospital Length of Stay	1.24 (0.54, 1.45)	1.32 (1.00, 2.00)	0.006
Discharge Category			0.7
Home	281 (84%)	69 (80%)	
Home Health	40 (12%)	12 (14%)	
I/P Rehab	12 (3.6%)	4 (4.7%)	
SNF Nursing Home	3 (0.9%)	1 (1.2%)	

^*1*^ Median (Q1, Q3); n (%)

^*2*^ Wilcoxon rank sum test; Pearson’s Chi-squared test; Fisher’s exact test

## Discussion

MIS lumbar spine surgery is an increasingly popular approach for surgical treatment of lumbar pathologies [40]. Unlike open approaches, which often require wide exposure and extensive paraspinal muscle dissection, MIS techniques preserve much of the muscle and fascia, potentially resulting in a lower physiological stress response to surgery and modifying the influence of systemic risk factors such as glycemic control [20]. The relatively lower degree of healing necessary for recovery in MIS approaches may also mitigate the effects of poor preoperative HbA1c. While the relationship between preoperative diabetes, elevated HbA1c, and postoperative complications have been extensively reported in the context of open spine surgery, the role of preoperative HbA1c optimization in MIS lumbar spine procedures remain unexplored [9, 11, 28]. Our results represents the first investigation of the association of preoperative HbA1c with increased readmissions and reoperations.

The results of DCCT trial demonstrated the importance of HbA1c control, with adequate control of HbA1c being associated with lower rates of diabetes associated complications [19]. Similarly, high HbA1c is associated with poor surgical outcomes in a variety of surgical specificalities. An analysis by Yong et al [39]. in a surgical inpatient sample at their institution found elevated HbA1c to be independently associated with increased odds of major complications, ICU admission, and longer hospital lengths of stay. Similarly, a meta-analysis by Wang et al [37]. suggested optimal HbA1c control is associated with lower rates of adverse events following surgery for coronary artery disease as well.

HbA1c has also been found to be associated with adverse outcomes in spine surgery. A multicenter analysis of the Michigan Spine Surgery Improvement Collaborative database highlighted that patients with HbA1c > 8.0% predicted increased complications, readmissions, and decreased functional improvement following lumbar spine surgery [31]. There also exists evidence of HbA1c as a superior predictor of lumbar spine outcomes compared to other measures of glucose control such as preoperative diabetes. An analysis by Hikata et al. [12] found high preoperative HbA1c, rather than glucose, to be significantly associated with SSI in a series of thoracolumbar fusion patients. Interestingly, our results did not find increased risk of infection. Though our study may be underpowered to investigate this particular outcome, it’s possible that the low exposure in MIS approaches may mitigate risk for infection.

However, despite the multitude of evidence as to the value of HbA1c optimization in open lumbar spine procedures, there exists a paucity of literature on optimal preoperative HbA1c for MIS approaches. Lower rates of infections, shorter lengths of stay, and greater benefits for patients with increased comorbidity burden are known advantages of MIS approaches, which may potentially minimize the negative effects of poorly managed preoperative HbA1c [18, 20]. Thus, it may be hypothesized that tight HbA1c control may not be as necessary for patients undergoing MIS procedures compared to those undergoing open procedures. To address this gap in the literature, we sought to investigate the outcomes of patients undergoing MIS lumbar spine procedures in those that had higher HbA1c. We found that HbA1c > 7.1% was independently associated with increased readmissions and infection/wound breakdown in the post operative period.

### Mechanisms

Poor glycemic control leads to cellular damage and organ failure, leading to poor wound healing and poor clinical outcomes in patients by way of immune and cellular dysfunction [25, 34]. Increased prolonged glucose is also associated with increased end-organ microvascular damage, leading to renal, retinal, and neurological breakdown and dysfunction [19]. High HbA1c was also associated with increased rates of reoperation at 90 days, consistent with open lumbar procedures.[29] Poor wound healing caused by poor HbA1c optimization would necessitate increased returns to the OR to revise wounds. Poor glycemic control is also known to be associated with poor bone health, leading to increased likelihood of instability, pathologic fractures, or other surgical complications following spine surgery, supporting our results of increased risk for reoperation for persistence of symptoms [38, 41]. This is supported by prior evidence demonstrating increased infections and poor functional outcomes in open lumbar spine surgery in patients with high HbA1c as well [29, 31].

High preoperative HbA1c was also associated with increased length of stay after adjustment for surgical complexity. Poor glycemic control may reflect an overall worse clinical condition, necessitating a longer recovery after lumbar spine surgery despite the reduced exposure necessary in MIS approaches. This hypothesis is similarly reflected in open lumbar spine surgeries as well, where analysis of patients undergoing surgery for adult spinal deformity (ASD) with increased HbA1c had prolonged hospital length of stay [34].

We also found that patients with poor glycemic control were more likely to be of low neighborhood socioeconomic status, represented by high ADI. Socioeconomic disadvantage is a well-established predictor of poor outcomes following lumbar spine surgery [7, 10, 17, 24, 27]. Thus, we sought to investigate the independent effects of both socioeconomic disadvantage and poor glycemic control in univariate and adjusted logistic regression models. High HbA1c remained as a significant predictor of poor outcomes, suggesting that perhaps poor glycemic control may be a mechanism by which socioeconomic status predicts poor post-operative outcomes in patients undergoing lumbar spine surgery. A multitude of literature exists highlighting the association of high neighborhood socioeconomic disadvantage and cardiovascular, diabetes, and comorbidity control [2, 6, 13, 15, 35]. However, whether or not poorly controlled HbA1c is a mechanism by which socioeconomic disadvantage portends poor spine surgery outcomes, though suggested by our analysis, serves as a potential direction of further investigation.

Our results serve as the first validation of HbA1c as a risk factor for increased adverse events in patients undergoing MIS lumbar spine surgery. Given the rising use of MIS approaches for lumbar spine surgery, along with the aging population and increasing rates of chronic diseases such as diabetes, it becomes increasingly important to understand how to best optimize risk factors in patients [23, 40]. Though the optimal cutoff for optimization should be explored in larger cohorts, our results demonstrate that HbA1c may be an important risk factor to consider in combination with psychosocial risk factors and clinical risk factors like frailty. Our results suggest that spine surgeons should seek to optimize HbA1c before MIS lumbar spine surgery to improve outcomes.

### Limitations

Our study is limited by its retrospective, single institution design, and thus the results may be limited in generalizability. Although we attempted to account for confounders, there may be surgical characteristics or unknown confounders that were not accounted for. Preoperative HbA1c, while, a popular metric, is not a perfect measure of diabetes control and may vary with anemic states. Additionally, HbA1c pre-operatively may not reflect post-operative HbA1c control, which may also play a significant role in patient outcomes. There is potential selection bias in the patient population, as patients with higher HbA1cs who received surgery may reflect a more acute indication. Though we only included elective cases and attempted to control for operative characteristics, some remaining degree of bias is still possible. The lack of patient-reported outcomes (PROs) such as ODI and VAS limit our analysis to objective measures, such as reoperation rates, and post-operative complications, which may not fully reflect the post operative course of patients. Future studies should prospectively evaluate the effect the of HbA1c on readmission rates and infection/wound issues. However, our results provide evidence to the value of preoperative HbA1c optimization for MIS lumbar spine surgery.

## Conclusion

Higher preoperative HbA1c is associated with increased readmissions, reoperations, complications, and length of stay following MIS lumbar spine surgery. Optimization of HbA1c prior to MIS procedures may improve outcomes.

## Supplementary Information

Below is the link to the electronic supplementary material.

ESM 1 (DOCX 326 KB)

## Data Availability

Data can be made available on reasonable request.

## Abbreviations

ADI: Area Deprivation Index
CMS: Centers for Medicare/Medicaid Services
HbA1c: Hemoglobin A1c
FIPS: Federal Information Processing Standard
HFRS: Hospital Frailty Risk Score
HRSA: Health Resources Services Administration
