# Supplementary material for: Preoperative hemoglobin A1c and minimally invasive lumbar spine surgery: is it as critical as we think
Source: Acta Neurochir (Wien). 2025 Nov 8;167(1):290. doi: 10.1007/s00701-025-06686-2 (PMC12596392; doi:10.1007/s00701-025-06686-2)
Supplement: Supplementary file 1 — (DOCX 326 KB) [file 701_2025_6686_MOESM1_ESM.docx]

**Supplementary Methods:**

*Variable Definition*

CPT codes utilized for query included 63005, 63017, 63042, 63044, 63047, 63048, 22558, 22585, 22612, 22614, 22630, 22633, 22634, 22842-22844. Patients with these codes were then manually reviewed for surgical characteristics. Body mass index (BMI) at time of surgery was collected from the EMR and categorized according to current convention (<18.5, underweight; 18.5 – 25, normal weight; 25-30, overweight; 30-35, Class 1 Obesity; 35-40, Class II Obesity; ≥ 40, Class III Obesity). ASA class at time of index surgery was assessed for comorbidity burden.

Operative characteristics such as vertebral levels involved and surgical approach (decompression with fusion vs decompression alone) were collected from the medical record and cross-referenced with billing codes. Post-operative complications included were defined as: durotomy/CSF leak, pneumonia, respiratory failure, sepsis, cerebrovascular accidence, DVT/PE, hematoma, delirium/acute mental status change, ileus, urinary incontinence, urinary tract infection, myocardial infarction, infection/wound breakdown, acute kidney injury, and hardware failure in a 90-day period after index operation.^1-4^ Post-operative variables included unplanned readmission within 90 days (POD 1-90) attributable to the index surgery. Reasons for readmission were categorized as readmission due to persistent pain or neurologic symptoms, readmission due to medical complications, and/or readmission due to infection.^5^ Reasons for reoperation were categorized as wound revision, persistent symptoms or instrumentation failure, wound revision, or other. Other outcomes included hospital length of stay, defined as total hospital length of stay for the surgical encounter.^6^ Discharge was categorized as home, home health, inpatient rehabilitation, and skilled nursing facility (SNF).^7^


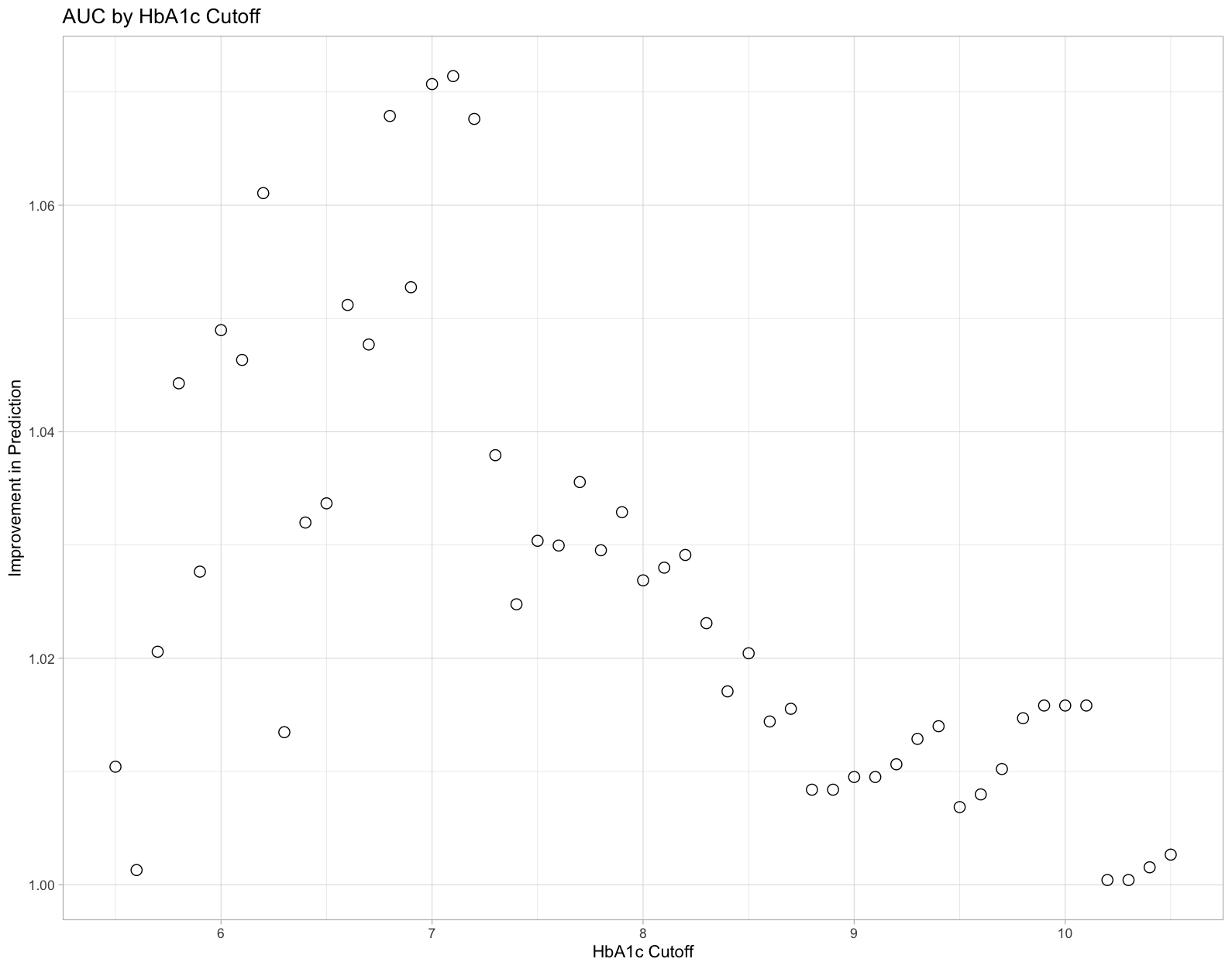


**Figure 1.** Identifying optimal cutoff for maximum predictive power for 90 day readmission


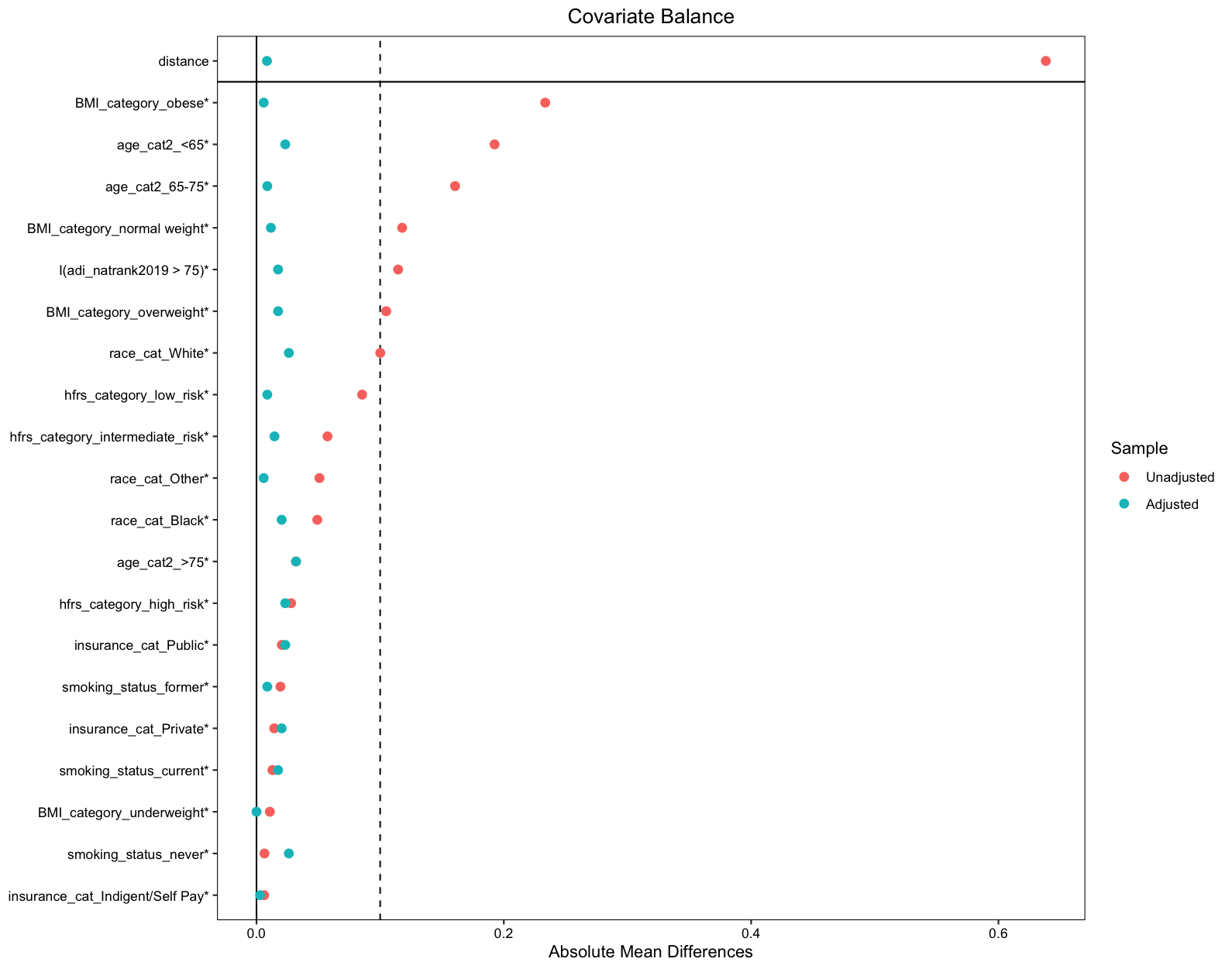


**Figure 2.** Love Plot for Matching Success

**Figure 3.** Imputation comparison for HbA1c

**Table 1.** VIF values assessing collinearity in readmission model

|  | GVIF | Df | GVIF^(1/(2*Df)) |
| --- | --- | --- | --- |
| preop HbA1C | 1.070426 | 1 | 1.034614 |
| Neighborhood Deprivation | 1.020758 | 1 | 1.010326 |
| Age | 1.40862 | 2 | 1.089428 |
| Insurance | 1.251953 | 2 | 1.057784 |
| Race | 1.086603 | 2 | 1.020981 |
| Frailty | 1.135845 | 2 | 1.032357 |
| Decompression vs Fusion | 1.013451 | 1 | 1.006703 |
| Smoking | 1.057368 | 2 | 1.014043 |
| BMI | 1.120088 | 3 | 1.019081 |

**Table 2.** VIF values assessing collinearity in 90 day reoperation model

|  | GVIF | Df | GVIF^(1/(2*Df)) |
| --- | --- | --- | --- |
| preop HbA1C | 1.058953 | 1 | 1.029054 |
| Neighborhood Deprivation | 1.02429 | 1 | 1.012072 |
| Age | 1.448614 | 2 | 1.09708 |
| Insurance | 1.355977 | 2 | 1.079103 |
| Race | 1.098464 | 2 | 1.023756 |
| Frailty | 1.125634 | 2 | 1.030029 |
| Decompression vs Fusion | 1.035601 | 1 | 1.017645 |
| Smoking | 1.113076 | 2 | 1.027144 |
| BMI | 1.119324 | 3 | 1.018965 |

**Table 3.** MissForest Imputation Diagnostic

| NRMSE | PFC |
| --- | --- |
| 2.277332e-06 | 3.335527e 02 |

**SUPPLEMENTAL REFERENCES**

1. Schoenfeld AJ, Ochoa LM, Bader JO, Belmont PJ, Jr. Risk Factors for Immediate Postoperative Complications and Mortality Following Spine Surgery: A Study of 3475 Patients from the National Surgical Quality Improvement Program. *JBJS*. 2011;93(17)

2. Chotai S, Parker SL, Sivaganesan A, et al. Effect of complications within 90 days on patient-reported outcomes 3 months and 12 months following elective surgery for lumbar degenerative disease. *Neurosurg Focus*. Dec 2015;39(6):E8. doi:10.3171/2015.8.Focus15302

3. Sivaganesan A, Zuckerman S, Khan I, et al. Predictive Model for Medical and Surgical Readmissions Following Elective Lumbar Spine Surgery: A National Study of 33,674 Patients. *Spine*. 2019;44(8):588-600. doi:10.1097/brs.0000000000002883

4. Glassman SD, Hamill CL, Bridwell KH, Schwab FJ, Dimar JR, Lowe TG. The Impact of Perioperative Complications on Clinical Outcome in Adult Deformity Surgery. *Spine*. 2007;32(24):2764-2770. doi:10.1097/BRS.0b013e31815a7644

5. Bernatz JT, Tueting JL, Anderson PA. Thirty-day readmission rates in orthopedics: a systematic review and meta-analysis. *PloS one*. 2015;10(4):e0123593.

6. Lee SY, Lee S-H, Tan JHH, et al. Factors associated with prolonged length of stay for elective hepatobiliary and neurosurgery patients: a retrospective medical record review. *BMC Health Services Research*. 2018/01/05 2018;18(1):5. doi:10.1186/s12913-017-2817-8

7. Mummaneni PV, Bydon M, Knightly JJ, et al. Identifying patients at risk for nonroutine discharge after surgery for cervical myelopathy: an analysis from the Quality Outcomes Database. *Journal of Neurosurgery-Spine*. Jul 2021;35(1):25-33. doi:10.3171/2020.11.Spine201442
